# *In Situ* EPR Studies of Reaction Pathways in Titania Photocatalyst-Promoted Alkylation of Alkenes

**DOI:** 10.3390/molecules20034055

**Published:** 2015-03-03

**Authors:** Shona Rhydderch, Russell F. Howe

**Affiliations:** Chemistry Department, University of Aberdeen, Aberdeen AB24 3UE, UK; E-Mail: shona.rhydderch@abdn.ac.uk

**Keywords:** EPR spectroscopy, titania, photocatalysis, alkene alkylation

## Abstract

*In situ* EPR spectroscopy at cryogenic temperatures has been used to observe and identify paramagnetic species produced when titania is irradiated in the presence of reactants used in the photocatalytic alkylation of maleimide with t-butyl carboxylic acid or phenoxyacetic acid. It is shown that maleimide acts as an acceptor of conduction band electrons. Valence band holes oxidise t-butyl carboxylic acid to the t-butyl radical and phenoxyacetic acid to the phenoxyacetic acid radical cation. In the presence of maleimide, the phenoxymethyl radical is formed from phenoxyacetic acid. The relevance of these observations to the mechanisms of titania photocatalyst-promoted alkylation of alkenes is discussed.

## 1. Introduction

Although semiconductor photocatalysis (SPC) is widely used in environmental applications for the destruction of organic compounds in solution or the gas phase [1], there is growing interest in applying the method to productive synthetic chemistry. The application of SPC to carbon-carbon bond forming reactions relies on utilizing free radicals generated by the reaction of organic molecules with valence band holes and/or conduction band electrons formed on band gap irradiation of an oxide semiconductor, usually titanium dioxide. For example, SPC additions of enol ethers to various acceptors have been described [2], as has the addition of tertiary amines to electron-deficient alkenes [3]. Cermenati *et al.* described trimethylsilyl derivatives as precursors for benzyl radical addition to electron-deficient alkenes under SPC conditions [4].

The photo-Kolbe reaction, first studied more than 30 years ago [5], involves the generation of alkyl radicals from simple aliphatic carboxylic acids using SPC with titania or metal-loaded titania catalysts. In this case, the reaction products were the corresponding alkanes and carbon dioxide. The wide variety of carboxylic acid precursors now available synthetically prompted us to revisit the photo-Kolbe reaction as a route to radicals, which may add to double bonds.

We recently reported the first successful application of this strategy to achieve alkylation and cyclisation reactions when a variety of carboxylic acid precursors reacted with electron-deficient alkenes in the presence of titania photocatalysts [6]. Subsequent papers have explored the structural features and functionality of the reactants, which are necessary and sufficient for productive chemistry [7] and have described the use of NMR spectroscopy to follow this chemistry in an NMR tube photoreactor [8].

EPR spectroscopy is a powerful technique for investigating radical formation in photocatalytic systems [9]. UV irradiation of suspensions of titania in the presence of t-BuCO_2_H in solution at room temperature produces transient signals of the t-Bu radical. Likewise, phenoxyacetic acid generates the PhOCH_2_ radical, vinylacetic acid the allyl radical and 2,2,2-triphenylacetic acid the triphenylmethyl radical [6,7]. The isotropic nature of the EPR signals observed from these radicals at room temperature suggested that they may be present in solution rather than on the titania photocatalyst surface. Their observation supports the reaction mechanisms postulated of radicals generated from the carboxylic acid precursors attacking the double bonds of electron-deficient alkenes, but does not address the issue of how radicals are generated at the photocatalyst surface.

This paper focusses on the use of low temperature (80 K or 4 K) *in situ* EPR spectroscopy to investigate the initial stages of the reaction pathways in two model SPC systems: the reaction of t-BuCO_2_H with maleimide and the reaction of phenoxyacetic acid with maleimide. In frozen solution at cryogenic temperatures, there is no opportunity for initially generated radicals to diffuse away from the titania surfaces. EPR spectroscopy then detects the initial products of the trapping of the valence band holes and conduction band electrons by adsorbed reactants during UV irradiation of the photocatalyst. We show that this provides further insight into the mechanisms of these reactions.

## 2. Results and Discussion

### 2.1. Blank Experiments with Acetonitrile Solvent

**Figure 1:**
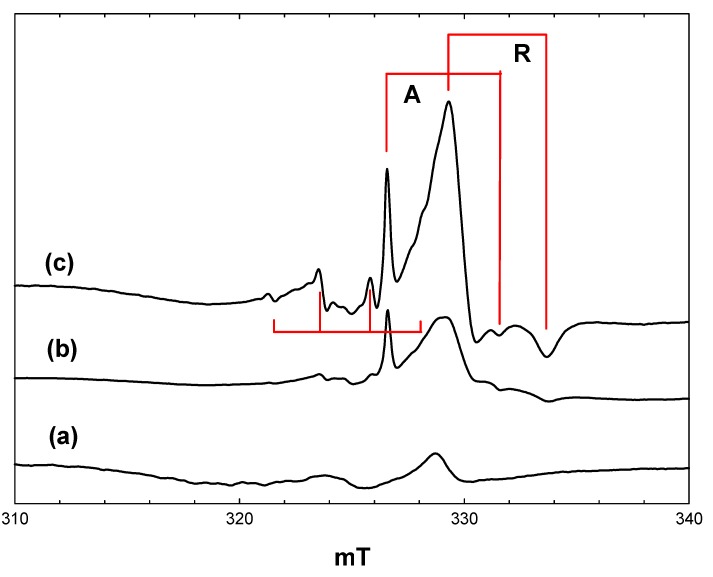
EPR spectra of P25 titania in acetonitrile at 4 K. (a), Prior to irradiation; (b), during irradiation with high intensity broad-band light; (c), after stopping irradiation.

The titania photocatalyst used in this work is Evonik P25, an approximately 70:30 mixture of anatase and rutile with a surface area of 50 m^2^·g^−1^. There is a substantial body of literature showing that this material has enhanced photocatalytic activity compared with either anatase or rutile, although there is not universal agreement as to the origins of this enhancement [10,11,12,13,14,15,16,17,18]. Figure 1 shows EPR spectra recorded at 4 K during and after irradiation *in situ* of a frozen suspension of P25 in dried and vacuum-outgassed acetonitrile. The initial spectrum prior to irradiation (Figure 1, a) shows only a very weak signal due to residual Ti^3+^ ions in the titania. On irradiation with high intensity broad band (320–900 nm) light, two intense signals appear (Figure 1, b), both due to Ti^3+^ ions resulting from trapping of conduction band electrons at Ti^4+^ sites. In the frozen suspensions, both signals show the line shapes characteristic of axial g-tensors. Comparison with the literature identifies the signals as Ti^3+^ in the anatase (g_perpendicular_ = 1.992, g_parallel_ = 1.962) and rutile (g_perpendicular_ = 1.973, g_parallel_ = 1.949) phases, respectively, marked as A and R in Figure 1.

Most authors have attributed the anatase signal to electrons trapped at interstitial Ti^4+^ defect sites [19,20], although this assignment has been challenged recently by Livraghi *et al.* [21]. They argue that the strictly axial symmetry and narrow line width of the signal are inconsistent with the distorted symmetry expected for interstitial species, while the presence of this species in many different types of doped and undoped anatase materials suggests that it is not associated with a particular type of defect. Analysis of the hyperfine interaction between this species and surrounding oxide ions (^17^O labelled) by pulsed EPR indicated that the Ti^3+^ signal is most likely due to d electrons delocalised over a number of adjacent lattice ions [21]. Regardless, this species is definitely associated with the anatase component of P25.

In the case of rutile, the signal seen in Figure 1 has parameters similar to those reported previously [22,23]. A definitive assignment of this signal to surface, bulk, lattice or interstitial Ti^3+^ does not seem to be possible from the literature, although evidence presented below suggests that it is probably associated with surface trap states in the rutile component of the P25.

When the irradiation was stopped at 4 K, there was an immediate marked increase in the intensity of both the rutile and anatase Ti^3+^ signals. (Figure 1, c). This is a consequence of the trapping of electrons, which are delocalised in the conduction band during irradiation. The number of trapped electrons detected during irradiation depends on the light intensity. At high light intensities, trapped electrons are re-excited into the conduction band, which competes with the trapping process, setting up a steady-state concentration of trapped electrons [22]. In the dark, electron trapping is the sole process occurring.

Also present in Figure 1, c, is a weak lower field quartet due to methyl radicals (hyperfine coupling of 2.3 mT). Experiments with acetonitrile-d_3_ showed that these do not originate from the acetonitrile, but are in fact due to traces of hydrocarbon contamination typically found in commercial titania photocatalysts. Valence band holes produced on irradiation are potent oxidizing agents and will readily generate methyl radicals from any adsorbed alkane.

The surprising feature of Figure 1 is the absence of any EPR signals due to trapped valence band holes to balance the number of trapped electrons detected. As suggested in [22], this is probably due to dimerization of hydroxyl radicals, which are the expected products of hole trapping at surface hydroxyl groups on the titania.

When a similar experiment to that described above was performed at 80 K instead of 4 K, the number of trapped electrons detected following irradiation in acetonitrile was lower, and in addition to the methyl radical (impurity) signal, a weak triplet due to the cyanomethyl radical ^.^CH_2_CN was observed. This showed the expected changes when acetonitrile-d_3_ was used. Since it was not formed at 4 K, it is probably resulting from H atom abstraction from CH_3_CN by initially formed methyl radicals at the higher temperature, rather than direct hole attack on acetonitrile.

### 2.2. Reaction of Pivalic Acid

**Figure 2:**
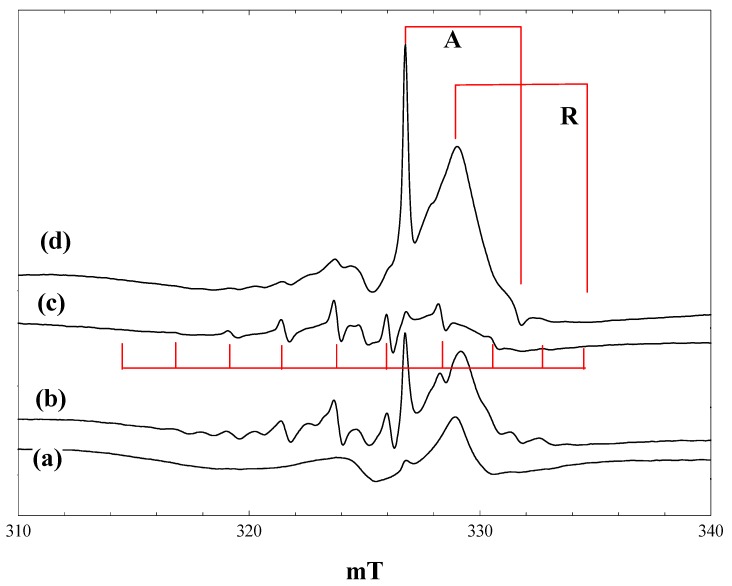
EPR spectra of P25 titania in a solution of pivalic acid in acetonitrile at 4 K. (a), During irradiation with low intensity 400 nm light; (b), during irradiation with high intensity 400 nm light; (c), during irradiation with high intensity broad band light; (d), after stopping irradiation.

Figure 2 shows EPR spectra obtained during irradiation at 4 K of a suspension of P25 in a 2.3 M solution of pivalic acid in acetonitrile. At low light intensity (1% of a 400 nm band pass filter), the only signals detected are those of the anatase and rutile Ti^3+^ trap states, plus a lower field signal due to trapped holes. The hole trapping process can be described as:

O^2−^ + h^+^ → O^−^

generating an odd electron species characterised by an axial g-tensor [24]. When the light intensity is increased (Figure 2, b), the trapped electron signals grow, along with a new 10-line signal of the t-butyl radical. On exposure to full intensity broad-band radiation, the trapped electron signals disappear, while the t-butyl radical signal remains unchanged (Figure 2, c). When the light is switched off, intense trapped electron signals in both the anatase and rutile phases reappear; the t-butyl radical signal is almost completely removed, and the trapped hole signal seen initially can be seen (Figure 2, d).

The anatase Ti^3+^ signal seen in the presence of pivalic acid is identical to that found in the blank experiment with solvent alone. The rutile signal, on the other hand, shows a small shift to a lower field in the perpendicular component (g_perpendicular_ = 1.976), while the g_parallel_ component is broadened and no longer sharply defined (g_parallel_ ~ 1.94). These observations confirm that the electron trap sites in anatase are in the bulk, whereas the rutile trap states are at the surface and influenced by the presence of adsorbed pivalic acid. Infrared spectra show clear evidence for both weakly bound molecular acid and more strongly bound carboxylate species when P25 is placed in contact with a solution of pivalic acid in acetonitrile. Binding of the carboxylic acid as a symmetrical carboxylate to surface hydroxyl groups bound to Ti^3+^ in the rutile phase would explain the changes in the Ti^3+^ EPR signal.

The 10-line t**-**butyl radical signal seen here has an apparent isotropic coupling constant of 2.25 mT, despite the fact that it is measured at 4 K. In alkyl radicals measured at low temperature, however, only hyperfine coupling from α protons shows a large anisotropy [25], and published data for the t-butyl radical in frozen solution at 4 K (A_parallel_ = 2.2 mT, A_perpendicul;ar_ = 2.4 mT [26]) confirm that the β proton coupling is near isotropic.

When the same experiment was performed at 80 K, two differences were noted. The number of trapped electrons detected after stopping irradiation was much lower than at 4 K, whereas the t-butyl radical signal was more intense and remained stable following irradiation at 80 K.

To account for these various observations, we consider the possible processes that can occur at the titania surface during and after irradiation. Electrons promoted into the conduction band may remain there during irradiation if the photon flux is sufficiently high [22], may be trapped at either bulk anatase or surface rutile trap states, may transfer to adsorbed molecules or recombine with valence band holes. The relative importance of each of these processes will depend on the photon flux, the temperature, the trapping efficiency of the trap states (which in the case of the surface trap states may be modified by the presence of adsorbed reactants) and the electron affinity and relative concentrations of adsorbed species. Holes generated in the valence band by 400 nm or broad-band irradiation may likewise remain free in the valence band during irradiation, be trapped at oxide ions as O^−^ (seen by EPR at low light intensities) or at surface hydroxyl groups as hydroxyl radicals (which are not seen by EPR, because of dimerization), accept electrons from adsorbed species, or recombine with conduction band electrons.

In the case of pivalic acid, the primary product of electron transfer is the t-butyl radical. The generation of this by hole attack can be written in one of three equivalent ways:

RCO_2_^−^ + h^+^ → R + CO_2_ or RCO_2_^−^ + O^−^ → R + CO_2_ + O^2−^ or RCO_2_^−^ + HO → R + CO_2_ + OH^−^

The R-CO_2_^−^ bond is broken in this step, but at 4 K, the CO_2_ product will remain in close proximity to the alkyl radical. When irradiation is stopped at 4 K, the t-butyl radical signal is lost. At 80 K, the yield of t-butyl radicals is higher, and the signal remains after irradiation. We suggest that at 80 K, the CO_2_ formed on breaking the R-CO_2_^−^ bond is able to diffuse sufficiently far from the t-butyl radical and that the reverse reaction:

R + CO_2_ + e → RCO_2_^−^

cannot occur. At 4 K, the reverse reaction, which corresponds to an electron: hole recombination, occurs when the irradiation is stopped. This explanation also accounts for the higher yield of t-butyl radicals at 80 K. In the absence of any other reactants, the electron trapping processes occur in a similar manner to that in the blank experiment with solvent alone.

### 2.3. Reaction of Phenoxyacetic Acid

**Figure 3:**
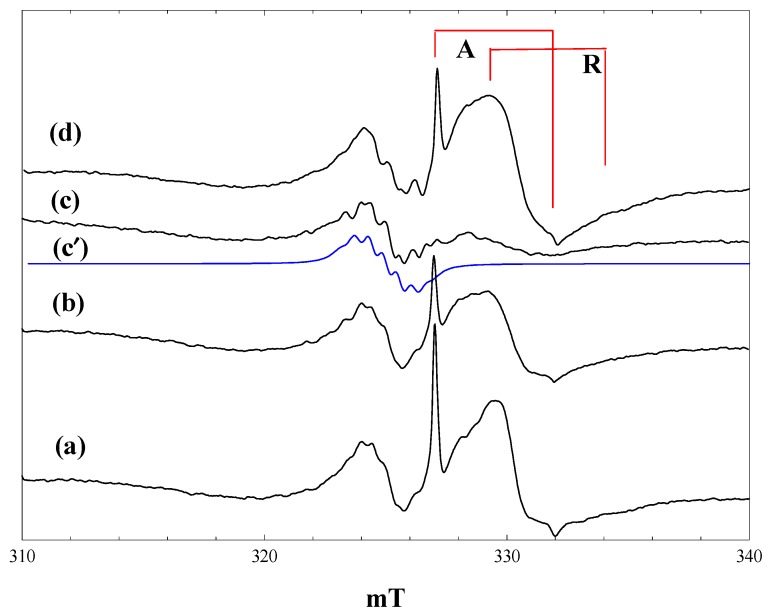
EPR spectra of P25 titania in a solution of phenoxyacetic acid in acetonitrile at 4 K. (a), During irradiation with low intensity 400 nm light; (b), During irradiation with high intensity 400 nm light; (c), during irradiation with high intensity broad band light; (d), after stopping irradiation; (c'), a simulated spectrum of the phenoxyacetic acid radical cation, as described in the text.

Figure 3 shows spectra measured during irradiation of a suspension of P25 in a 2.3 M solution of phenoxyacetic acid in acetonitrile at 4 K. Initial irradiation with low intensity 400 nm light gave already intense signals of trapped electrons in anatase and rutile, plus a new signal centred at g = 2.002, showing a poorly resolved hyperfine structure (Figure 3, a).

The trapped electron signals decreased when the intensity of the 400-nm light was increased (Figure 3, b), and they were almost completely removed when high power broad-band irradiation was performed (Figure 3, c). These signals reappeared when the irradiation was stopped (Figure 3, d). The new low field signal, which appeared on initial low intensity irradiation, remained at the same intensity throughout, although it did show some improved resolution when the light intensity was increased.

The new signal was much less evident when the same experiment was performed at 80 K, and it was completely destroyed when the temperature was raised above 120 K. We have considered several possible assignments for the new signal. It is very clearly not the phenoxymethyl radical, which would be the equivalent of the alkyl radicals formed from aliphatic carboxylic acids. This has been detected in solution at room temperature and gives a spectrum comprising a simple triplet with an isotropic proton coupling of 1.74 mT [6]. The hyperfine splitting seen in Figure 3 is much smaller than this and comprises at least seven lines. An alternative possibility is that valence band holes oxidise the aromatic ring to form a radical cation in which the unpaired electron is coupled to the aromatic ring protons. Infrared spectra of phenoxyacetic acid adsorbed on P25 show that it is present as both the weakly bound molecular species and the more strongly bound carboxylate anion; we suggest that it is the anion that acts as a trap for valence band holes. Note also that the trapped electron signal from the rutile phase shows similar differences from that seen with acetonitrile alone to those found with pivalic acid, further evidence for the adsorption of the carboxylic acid on the surface. No hyperfine coupling data have been reported in the literature for the radical cation of phenoxyacetic acid, but hyperfine coupling constants are available for the radical cation of anisole [27]: methyl protons (three), 0.483 mT; ring protons 0.452, 0.021, 0.997, 0.100 and 0.551 mT. We attempted to simulate the observed spectrum assuming that the aromatic ring protons in the phenoxyacetic acid cation would have similar coupling constants as those of the anisole cation and that the methylene proton coupling would be the same magnitude as the methyl coupling in the anisole cation (two protons instead of three). The coupling constants were then adjusted to best match the experimental spectrum. The simulated spectrum shown in Figure 3, c*'*, was obtained with the following (isotropic) coupling constants: methylene protons (two), 0.58 mT; ring protons 0.55, 0.65 and 1.10 mT (the two smallest proton couplings in the anisole cation were neglected, given that they were much smaller than the observed line width).

The observed spectrum is not well enough resolved to allow a high level of confidence in the precise values of the coupling constants (attempts to improve resolution by varying microwave power and modulation amplitude were unsuccessful). A second factor contributing to the line width and the difficulty of simulation is the presence of anisotropy in the hyperfine coupling. The para proton in the toluene radical cation shows a large hyperfine anisotropy [28], and this would be expected also in the phenoxyacetic acid radical cation. Nevertheless, we believe that the reasonable level of agreement between the observed and simulated spectra in Figure 3 supports our identification of the aromatic radical cation as the primary product of hole trapping from phenoxyacetic acid at 4 K. 

The aromatic radical cation is stable at 4 K in the absence of any other reactants. In particular, at this temperature, there is no cleavage of the RCH_2_-CO_2_^−^ bond to form the benzyl radical. At 80 K, much less of the radical cation is seen, due to more facile back transfer of an electron from the titania at this temperature.

### 2.4. Reaction of Maleimide

**Figure 4:**
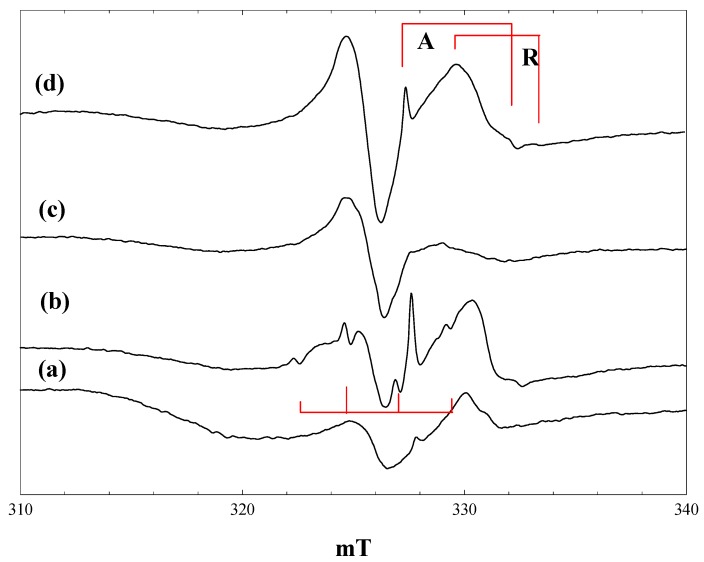
EPR spectra of P25 titania in a solution of maleimide in acetonitrile at 4 K. (a), During irradiation with low intensity 400 nm light; (b), during irradiation with high intensity 400 nm light; (c), during irradiation with high intensity broad band light; (d), after stopping irradiation.

Figure 4 shows spectra measured during irradiation of a suspension of P25 in a 2.3 M solution of maleimide in acetonitrile at 4 K. At low light intensities, signals of trapped electrons in anatase and rutile are detected, along with the lower field signal of trapped holes. With full light intensity at 400 nm, traces of methyl radicals from organic contaminants in the P25 are detected (Figure 4, b), but these are no longer seen during full broad-band irradiation (Figure 4, c). The dominant signal seen at this stage is a near isotropic line at g = 2.002. When the light is turned off, this signal grows in intensity, along with the Ti^3+^ signals of trapped electrons in anatase and rutile (Figure 4, d). These trapped electron signals are, however, much reduced in intensity compared with those with acetonitrile alone (Figure 1) or with the carboxylic acids.

Radiolysis of aqueous solutions of maleimide gives EPR spectra of the maleimide radical anion, which is characterised by hyperfine coupling from ^14^N (0.262 mT), the two olefinic protons (0.580 mT) and the NH proton (0.031 mT) [29]. Given the much reduced yield of trapped electrons formed when P25 is irradiated in the presence of maleimide, we suggest that the maleimide is acting as an electron scavenger, forming the maleimide radical anion.

Similar spectra were measured when the same experiment was performed at 80 K, although the trapped electron signals were markedly less intense at this temperature and not detected at all following broad-band irradiation. The maleimide radical anion signal was destroyed by raising the temperature to 160 K.

**Figure 5:**
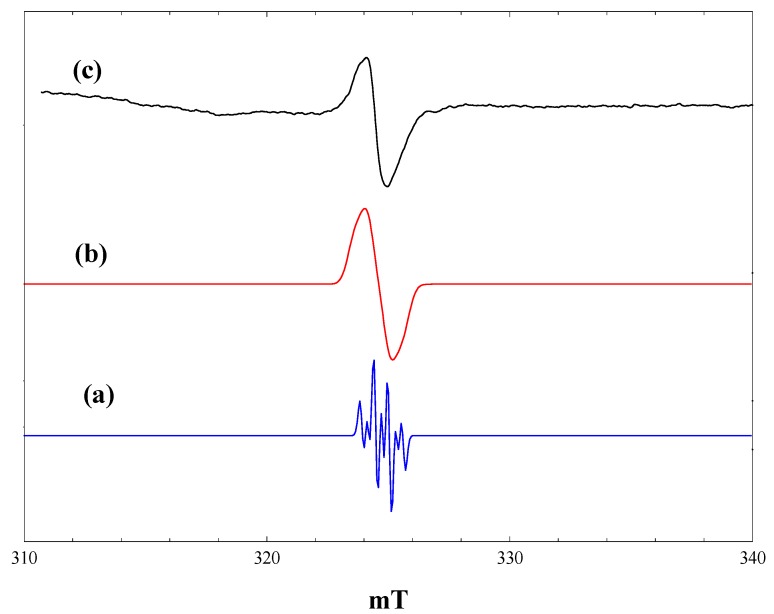
Comparison of simulated and observed spectra of the maleimide radical anion. (a), Simulated with a(N) = 0.262 mT, a(2H) = 0.580 mT, a(NH) = 0.031 mT, Gaussian line width of 0.2 mT; (b), Gaussian line width of 0.6 mT; (c), observed spectrum following broad-band irradiation at 80 K.

In the absence of well-resolved ^14^N or ^1^H hyperfine splitting in the signal, the assignment to the maleimide radical anion remains tentative. Nevertheless, the observed line shape could be satisfactorily simulated with the isotropic hyperfine couplings for the radical anion taken from [29] and a Gaussian line width of 0.6 mT. The large linewidth may reflect some degree of anisotropy in the hyperfine couplings. Figure 5 compares the simulated spectra obtained with line widths of 0.2 mT, 0.6 mT and the experimentally-observed signal (at 80 K). Formation of the radical anion greatly reduces the number of conduction band electrons that are trapped in the titania.

### 2.5. Reaction of Pivalic Acid with Maleimide

**Figure 6:**
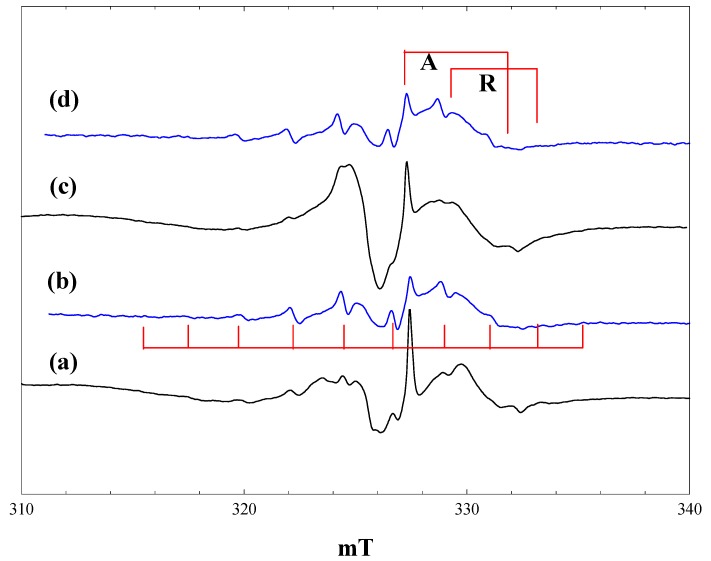
EPR spectra of P25 titania in an equimolar mixture of maleimide and pivalic acid in acetonitrile. (a), During irradiation with high intensity 400 nm light at 4 K; (b), during irradiation with high intensity 400-nm light at 80 K; (c), after irradiation with high intensity broad-band light at 4 K; (d), after irradiation with high intensity broad band light at 80 K.

The titania photocatalysed reaction of pivalic acid with *N*-phenyl maleimide is reported to give approximately equal yields of the alkylated addition product and *N*-phenyl succinimide [7]. To model this chemistry, we chose to examine by *in situ* EPR the initial paramagnetic species formed when an equimolar mixture of pivalic acid and maleimide was irradiated in the presence of P25 at 4 K and at 80 K. Figure 6 shows the spectra observed.

Irradiation of the mixture with full intensity 400-nm light at 4 K produced both the t-butyl radical and maleimide radical anion signals seen in the separate experiments (Figure 6, a), although the yields of both were lower with the mixture. (Compare Figure 6, a, with Figure 2, b, and Figure 4, b). The signal due to electrons trapped at (bulk) anatase sites had comparable intensity to that seen with pivalic acid alone, whereas the number of electrons trapped at (surface) rutile sites was much lower.

After full intensity broad-band irradiation at 4 K (Figure 6, c), the t-butyl radical signal was almost completely removed, leaving a strong signal of the maleimide radical anion. The intensity of the trapped electron signals in this case was much less than those seen with pivalic acid alone (Figure 2, d). When the same experiment was performed at 80 K, on the other hand (Figure 6, d), the t-butyl radical remained following broad-band irradiation, whereas the maleimide radical anion was almost completely removed.

In the presence of maleimide, the yield of t-butyl radicals at 4 K is reduced, possibly because of competition between pivalic acid and maleimide for surface sites on the titania. Likewise, the yields of maleimide radical anions at both 4 K and 80 K are lower, and the number of trapped electrons higher in the presence of pivalic acid. The formation of succinimide in parallel with the addition product of the t-butyl radical to maleimide in the room temperature photocatalytic reaction [7] can be attributed to a further one-electron reduction of the maleimide radical anion (accompanied by protonation).

### 2.6. Reaction of Phenoxyacetic Acid with Maleimide

**Figure 7:**
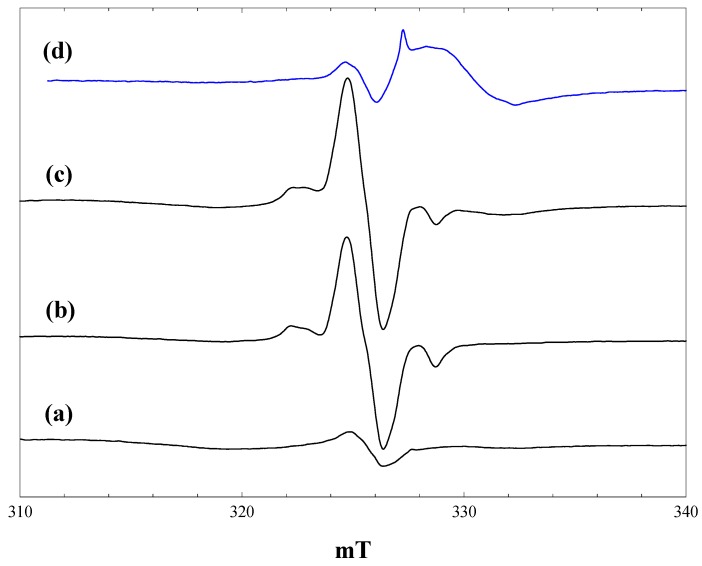
EPR spectra of P25 titania in an equimolar mixture of maleimide and phenoxyacetic acid in acetonitrile. (a), during irradiation with high intensity 400 nm light at 4 K; (b), during irradiation with high intensity broad band light at 4 K; (c), after irradiation with high intensity broad band light at 4 K; (d), after irradiation with high intensity broad band light at 80 K.

Irradiation of equimolar mixtures of phenoxyacetic acid and maleimide in the presence of P25 at 4 K gave spectra that were strikingly different from those obtained with the separate reactants. Figure 7 shows the spectra obtained.

Very few trapped electrons are detected at any stage during or after irradiation at 4 K. The central feature of the spectra in Figure 7, b and c, may contain a contribution from the maleimide radical anion, but the features at ~3.2 mT below and above this feature were not seen in the spectra obtained with phenoxyacetic acid or maleimide alone. At 80 K, on the other hand, the spectrum obtained following broad-band irradiation (Figure 7, d) is similar to that obtained with phenoxyacetic acid alone, showing the phenoxyacetic acid radical cation and trapped electrons.

The phenoxyacetic acid radical cation will be the primary product of electron transfer from adsorbed phenoxyacetic acid:

PhOCH_2_CO_2_^−^ + h^+^ → [PhOCH_2_CO_2_^−^]^+^

and this is the process seen when phenoxyacetic acid is irradiated alone at 4 K or 80 K. The radical cation is the precursor of the phenoxymethyl radical, which was not detected when phenoxyacetic acid was irradiated alone:

[PhOCH_2_CO_2_^−^]^+^ → PhOCH_2_^.^ + CO_2_

The extent to which CH_2_-CO_2_^−^ bond scission occurs will depend on the stability of the radical cation *vs.* the reversal of its formation through electron transfer from the conduction band or from trap sites. We suggest that in the presence of maleimide, which is an electron scavenger, the back reaction is disfavoured, and the phenoxymethyl radical is formed at 4 K.

**Figure 8:**
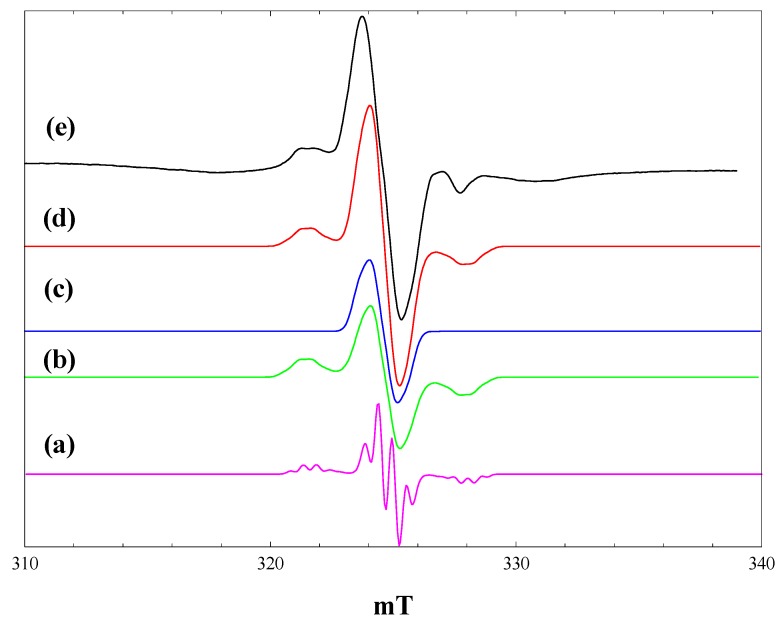
Simulation of the observed spectrum obtained during irradiation of P25 titania in an equimolar mixture of maleimide and phenoxyacetic acid in acetonitrile with high intensity broad-band light at 4 K. (a), Simulation of the phenoxymethyl radical using the parameters described in the text and Gaussian line widths of 0.3 mT (parallel) and 0.5 mT (perpendicular); (b), Gaussian line widths of 0.5 mT (parallel) and 1.0 mT (perpendicular); (c), simulation of the maleimide radical anion (from Figure 5, b); (d), Sum of b and c; (e), observed spectrum.

The spectra in Figure 7, b and c, can be simulated as the superposition of the maleimide radical anion and the phenoxymethyl radical. This process is illustrated in Figure 8. Isotropic hyperfine coupling constants have been reported for the phenoxymethyl radical formed by laser photolysis of anisole in solution [30]. To simulate the phenoxymethyl radical spectrum, the aromatic proton coupling constants were taken as the isotropic values reported in [30] (neglecting the smallest meta-proton values of 0.27 mT), while the CH_2_ coupling was assumed to be axially symmetric with an averaged isotropic value equal to that in solution (1.7 mT).

Figure 8, a, shows a simulated spectrum for the phenoxymethyl radical with the following parameters: g_iso_ = 2.002, A_parallel_(CH_2_) = 3.2 mT, A_perpendicular_(CH_2_) = 0.9 mT, A_iso_(2H) = 0.54 mT, A_iso_(1H) = 0.49 mT, Gaussian line widths of 0.3 mT (parallel) and 0.5 mT (perpendicular). The anisotropy in the CH_2_ hyperfine coupling (A_parallel_ − A_perpendicular_ = 2.1 mT) is comparable to that seen in the 2-hexyl radical (~2.0 mT) [25]. When the line-widths in the simulation are increased to 0.5 mT (parallel) and 1.0 mT (perpendicular) (Figure 8, b) and an equal contribution added to form the simulated spectrum of the maleimide radical anion (Figure 8, c), the resulting simulated spectrum matches well with the observed spectrum.

The phenoxymethyl radical is not seen when the phenoxyacetic acid:maleimide mixture is irradiated at 80 K (Figure 7, d). This is attributed to the enhanced rate of back electron transfer at 80 K, disfavouring the bond cleavage reaction.

## 3. Experimental Section

All reagents were purchased from Sigma-Aldrich: acetonitrile (99.8%), t-butyl carboxylic acid (99%), phenoxyacetic acid (99%) and maleimide (99%). P25 titania was purchased from Evonik.

EPR measurements were carried out using a JEOL JES-FA200 spectrometer, with an X band microwave unit (≈9 GHz). Power levels of between 0.5 and 5 mW were used. Temperature control was achieved with an Oxford Instruments ESR900 cryostat, with liquid nitrogen for temperatures down to 77 K, or liquid helium for temperatures down to 4 K. Cavity temperatures were measured using a Lakeshore CX1050 Cernox sensor, located 2 mm below the sample.

Samples were prepared by placing a solution of the organic substrate (0.5 mL, 2.3 M in acetonitrile) in a side arm, sealed by a Young’s tap, attached to a 5-mm quartz EPR tube fitted with a second Young’s tap. Forty milligrams of Evonik P25titania were placed in the EPR tube and air and physisorbed water removed by attaching the cell to a vacuum line and degassing overnight (10^−5^ torr) at room temperature. The organic solution was degassed by repeated freeze pumping with liquid nitrogen. The sample cell was then sealed, removed from the vacuum line and the organic solution carefully decanted onto the titania by opening the Young’s tap.

Samples were irradiated *in situ* using a 450-Watt LOT Hg-Xe arc lamp, filtered through Pyrex and water filters to remove the UV (<320 nm) and IR (>900 nm) components of the light, respectively. The light intensity was reduced as needed with neutral density filters and/or a 400-nm band pass filter (60-nm half-width) and the output focused through a lens into the EPR cavity. Light intensities were calibrated with an Optronic Laboratories OL750 automated spectroradiometric measurement system. In the spectra presented above, high intensity broad-band irradiation (320–900 nm) was measured to be 308 mW·m^−2^; high intensity 400-nm irradiation 28 mW·m^−2^; and low intensity 400-nm irradiation 0.28 mW·m^−2^.

Simulation of the EPR spectra was performed using the program, SIM32 [31].

## 4. Conclusions 

These *in situ* EPR experiments have clarified a number of issues concerning the reaction pathways in the photocatalytic reactions of carboxylic acids with electron-deficient alkenes. The species observed are in all cases the initial products of electron or hole transfer at the catalyst surface. Hole attack on adsorbed pivalic acid, like acetic acid [5], cleaves the R-CO_2_^−^ bond to generate alkyl radicals, but the reaction is reversed at 4 K when illumination is stopped. In the case of phenoxyacetic acid, the primary product of hole attack is the adsorbed phenoxyacetic acid radical cation, which does not undergo bond cleavage at 4 K, unless maleimide is present to scavenge electrons. Maleimide readily accepts electrons at 4 K to form the radical anion. All of these processes are in competition with electron trapping in the titania (seen directly as Ti^3+^) and hole trapping (some O^−^ detected by EPR, but mostly dimerised and EPR invisible). The relatively inefficient alkylation of alkenes with simple aliphatic carboxylic acids reported in [7] compared with acids yielding benzyl radicals is consistent with the EPR observations of higher radical yields (in the presence of the electron scavenger) from phenoxyacetic acid than from pivalic acid.

The photocatalytic synthesis reactions with these molecules are all performed at room temperature, whereas none of the adsorbed species detected in the EPR experiments described here persist above about 120 K. Nevertheless, the observation of these and related radicals during steady-state irradiation in the solution phase at room temperature [6,7] makes it likely that they are generated by the desorption of the initially-formed adsorbed species from the photocatalyst surface into solution. An unresolved question is the extent to which the radical addition reactions occur on the catalyst surface (the so-called direct process) or between desorbed radical species in the liquid phase (the so-called indirect process) [32,33].
